# Assessment of Blue Carbon Storage by Baja California (Mexico) Tidal Wetlands and Evidence for Wetland Stability in the Face of Anthropogenic and Climatic Impacts

**DOI:** 10.3390/s18010032

**Published:** 2017-12-24

**Authors:** Elizabeth Burke Watson, Alejandro Hinojosa Corona

**Affiliations:** Departamento Geología, Centro de Investigación Científica y de Educación Superior de Ensenada, 22860 Ensenada, BC, Mexico; alhinc@cicese.mx

**Keywords:** climate change, salt marsh, mangroves, aeolian, sediment transport, marsh loss, coastal development, remote sensing, carbon stable isotopes

## Abstract

Although saline tidal wetlands cover less than a fraction of one percent of the earth’s surface (~0.01%), they efficiently sequester organic carbon due to high rates of primary production coupled with surfaces that aggrade in response to sea level rise. Here, we report on multi-decadal changes (1972–2008) in the extent of tidal marshes and mangroves, and characterize soil carbon density and source, for five regions of tidal wetlands located on Baja California’s Pacific coast. Land-cover change analysis indicates the stability of tidal wetlands relative to anthropogenic and climate change impacts over the past four decades, with most changes resulting from natural coastal processes that are unique to arid environments. The disturbance of wetland soils in this region (to a depth of 50 cm) would liberate 2.55 Tg of organic carbon (C) or 9.36 Tg CO_2_eq. Based on stoichiometry and carbon stable isotope ratios, the source of organic carbon in these wetland sediments is derived from a combination of wetland macrophyte, algal, and phytoplankton sources. The reconstruction of natural wetland dynamics in Baja California provides a counterpoint to the history of wetland destruction elsewhere in North America, and measurements provide new insights on the control of carbon sequestration in arid wetlands.

## 1. Introduction

Seagrass beds, mangrove forests, and salt marshes are thought to cover 330,000 to 1,150,000 km^2^ globally [1], less than a fraction of one percent of the earth’s surface (0.007–0.02%). Yet they are efficient at organic carbon (C) sequestration due to high rates of primary production [2] and soils that do not become saturated in C. Rather, healthy vegetated coastal ecosystems aggrade vertically in response to sea level rise [3], which sequesters a combination of autochthonous C produced in situ as plant growth by rooted macrophytes, and allochthonous C intercepted from watersheds and estuaries as particulate organic carbon (POC) [4], buried as mineral sediment accumulates. The burial of organic C in saline wetland soils in coastal wetlands and seagrass beds (‘blue carbon’) has been estimated at ~100 Tg C year^−1^, 10 times greater than deep sea C burial [5,6].

Vegetated coastal ecosystems are additionally important as global C sinks, as their net global warming potential is not reduced by significant emissions of the potent greenhouse gas methane, which has a considerably higher (25 times) global warming potential than carbon dioxide on a 100-year time horizon [7]. While the net climate forcing of freshwater wetlands is thought be positive due to methane emissions [8], at soil salinities above 18‰, bacteria that mineralize organic C in concert with sulfate reduction outcompete the methanogenic bacteria that decompose C anaerobically and produce methane [9,10]. Thus, methane emissions are typically low where soil salinities are high [11,12]. Based on high C sequestration rates and negligible emissions of methane, recent analyses conclude that vegetated coastal habitats have a strong net benefit for reducing global warming [11,13].

The global blue C sink is being diminished by anthropogenic activities and climatic change. A decline in the areal extent of vegetated coastal habitats is being driven by coastal development; the dredging, diking, and filling of coastal wetlands; and eutrophication, which is harmful for seagrass beds. Estimates of current declines in the global blue C sink range from 0.7% to 7% annually [1,6]. Although countries may require habitat mitigation where development impacts coastal ecosystems [13], declines in seagrass beds and coastal wetlands are expected to continue as accelerated sea level rise drives reductions in coastal wetland extent [14], higher temperatures increase the mineralization of sequestered C [15], and nutrient pollution intensifies as the expanding global population concentrates in coastal cities [14]. The global importance of the blue C sink combined with concerns regarding high rates of coastal vegetation loss justifies the investigation of regionally significant areas of coastal wetlands to determine C inventories and current loss trajectory.

Previous studies have focused on establishing wetland change rates over time and on determining country or habitat-level coastal wetland C stocks. Long-term wetland change has been calculated by comparing contemporary and historic wetland distribution patterns based either on historic maps [16,17] or potential distributions estimated using soil and topographic data [18,19]. For more recent time periods, wetland change has been calculated using aerial photography or satellite imagery [20]. Stocks and rates of C sequestration are calculated using an inventory of sediment cores analyzed for organic C density collected from the environments under consideration [21,22,23]. Although previous work has focused on both wetland change [24] and C sequestration [22] in Baja California, past studies have not considered the full holistic context necessary to manage C stocks for sustainability. This includes information on past and future losses to coastal development or disturbance, as well as natural geomorphological processes that lead to wetland creation and destruction [25]. Information on responses that are occurring due to climate change, including losses due to drowning [26] or increases due to upslope migration [24] are relevant, as well as assessments of sediment source and potential disruptions to sediment transport pathways that may threaten wetland survival [27]. Lastly, understanding the source of C preserved in wetland sediments, as well as interactions with climatic and environmental gradients, can provide important insights into the coastal C cycle [23].

The objectives of this study were to produce a preliminary assessment of C sequestration in the upper horizons of coastal wetland soils for the Baja California peninsula, and to determine whether changes in C sequestration are resulting from modifications to wetland extent caused by coastal development or climate change [24,28]. The lagoons and estuaries of the Baja California peninsula support a sizeable and pristine expanse of coastal wetlands, which include salt marshes of unusually high genetic and floristic diversity [29], and south of 28 degrees, a mosaic of tidal wetlands and shrub mangroves [24]. To establish the extent of coastal wetlands for C sequestration calculations and to detect changes in wetland extent related to coastal development or climate change impacts, tidal wetland vegetation boundaries were digitized using 1972 and 2008 aerial imagery for the five largest estuary/lagoon complexes along the Pacific coast of the Baja California peninsula. Soil C storage was quantified through measures of C density obtained from sediment cores collected from the same five areas of wetlands, and C source analysis was performed using stoichiometric and stable isotope ratios. Additional geochemical and particle size distribution measurements were performed on wetland, dune, and fluvial sediment samples to identify sediment sources. Measures of C storage by Baja California’s tidal wetlands, reported here, provide new insights on the control of C sequestration in arid tidal wetland soils.

## 2. Materials and Methods

### 2.1. Environmental Setting

The climate of the Baja California peninsula is classified as arid, with a mean annual temperature on the Pacific Coast of 22 °C, a January mean temperature of 14–16 °C, and an August mean temperature of 22–29 °C. Precipitation is ~150 mm year^−1^, and occurs during winter mid-latitude cyclonic activity and during summer convection storms and infrequent tropical storms and cyclones. Precipitation seasonality grades shift from Mediterranean (dry summer) in the north to North American Monsoonal (wet late summer) in the south. Lagoons are hypersaline inverse estuaries, with a typical salinity range of 34–39‰ [30,31]. The tidal regime is semidiurnal mixed; the tidal range is 1–2.5 m.

**Figure 1:**
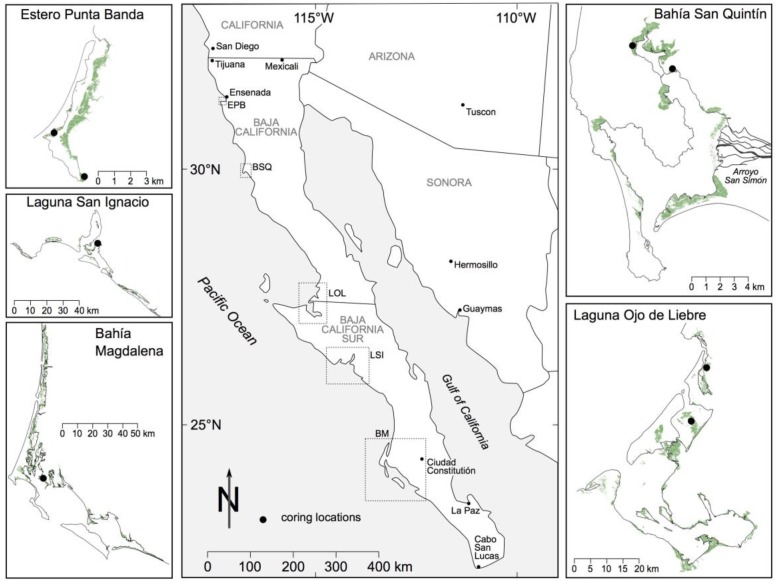
Location of study region. Inset maps show the distribution of tidal wetlands, in green, for each estuary/lagoon complex. Locations of sediment core samples are indicated.

The five largest areas of tidal wetlands found on the Baja California peninsula were identified based on spatial inventories developed by Pro Esteros, a Mexican conservation NGO based in Baja California. Focus sites include: Estero Punta Banda (EPB), Bahía San Quintín (BSQ), Laguna Ojo de Liebre (LOL), Laguna San Ignacio (LSI), and Bahía Magdalena (BM); all are tectonic estuaries protected by coastal barriers (Figure 1). Four of the five lagoons are Ramsar sites (Wetlands of International Importance); two (Laguna San Ignacio and Laguna Ojo de Liebre) form part of the El Vizcaíno Biosphere Reserve, México’s largest protected area, and a Unesco World Heritage site. The three southern lagoons are nurseries for the eastern subpopulation of the North Pacific gray whale, the International Union for the Conservation of Nature (IUCN) red-listed green turtle (*Chelonia mydas*) and loggerhead turtle (*Caretta caretta*). All five estuaries support extensive eelgrass populations, and are regionally significant overwintering grounds for waterfowl and shorebirds [32,33].

### 2.2. Carbon Storage

#### 2.2.1. Remote Sensing

To establish a contemporary estimate of tidal wetland areal extent, and to identify changes in wetlands over time, wetland vegetation boundaries at study sites were photo-interpreted using aerial photographs from 1972 and high-resolution satellite imagery circa 2008. The 2008 imagery was downloaded as tiles from Google Earth Pro as color digital orthoimagery (Google, Digital Globe). Spatial referencing was preserved by saving associated world files (e.g., jpgw), which record the location, scale, and rotation of the imagery. The 1972 imagery was obtained from INEGI (Instituto Nacional de Estadística y Geografía, Aguascalientes, Mexico) as color and B/W prints with scales ranging from 1:25,000 to 1:70,000, which were scanned at 500 dpi. The 1972 imagery was georeferenced in ArcGIS, version 9.1 using the 2008 orthoimagery as a base map. To ensure that changes detected were not a function of differing resolution, imagery was resampled to produce identical pixel dimensions (2.5 m), and the color imagery was rendered in grayscale using a red–green–blue to hue-saturation-intensity conversion, with the intensity component interpreted. Wetlands were digitized as a raster overlay using the remote sensing software ENVI (Exelis Visual Information Solutions, Boulder, CO, USA) by employing a semi-automated procedure that used scene-specific operator-defined intensity thresholds to classify imagery [34]. Site visits were made to verify wetland boundaries. Classification performance was evaluated through comparison with heads-up digitization [35] conducted at 50 randomly chosen (10 each per estuary) 1-km circular polygons, which included multiple land covers (e.g., wetlands, open water, dunes). Where wetland loss or gains were prevalent, imagery was examined to identify mechanisms.

#### 2.2.2. Sedimentary Analyses

Sediment cores with a length of 50–200 cm were collected from each lagoon using a Russian peat borer, which is a side filling coring device that collects sediment samples free of compaction (Aquatic Research Instruments, Hope, ID, USA). Cores were X-rayed at the University of California Davis, School of Veterinary Sciences, and sub-sampled at 2-cm intervals for bulk density and organic content determinations. Density and organic content measures were made using loss on ignition (LOI) by sub-sampling a known volume of sediment, drying it to a constant weight, re-weighing, ashing the sub-sample at 550 °C for four hours, and measuring a final weight [36]. For samples collected from upper layers (0–50 cm, *n* = 75), sediments were analyzed in duplicate for organic C, nitrogen composition and C stable isotope ratios using a Vario Micro Cube elemental analyzer coupled to an Isoprime stable isotope ratio mass spectrometer (IRMS). Samples were pretreated using hydrochloric acid fumigation prior to analysis to remove inorganic C. As soil organic C was a strong linear function of LOI values (*r*^2^ = 0.95, *p* < 0.01), organic C estimates were produced using LOI values for samples not analyzed for organic C. Soil C composition (stable isotope and OC:N stoichiometric ratios) was compared with potential C source end-members, including phytoplankton; macropyhte plant tissue from *Ulva* (an opportunistic macroalgae); *Zostera marina*; C3 and C4 marsh plants; and mangroves from Bahia San Quintin [37], the Tijuana Estuary and the San Dieguito Lagoon [38], San Francisco Bay [3,39], and the Yucatan Peninsula [40].

Sediment sub-samples were collected from 10-cm intervals and analyzed for particle size distribution and geochemistry. Multiple aliquots of heated hydrogen peroxide were used to oxidize organic material, and sodium hexametaphosphate was used as a dispersant [41], prior to introduction into a Beckman-Coulter LS230 laser granulometer (Beckman-Coulter Inc., Fullerton, CA, USA). Particle size central tendency was reported as inclusive graphic mean (in μm) [32] and sorting as inclusive graphic standard deviation (in Φ; values <0.5 Φ are well-sorted samples, 0.5–1.0 Φ moderately sorted, >1.0 Φ poorly sorted, and >2.0 Φ very poorly sorted). Sub-samples were analyzed for bulk geochemistry using a four-acid digestion method followed by analysis with an inductively coupled plasma atomic emissions spectrometer (ICP-AES). Potential mineral sediment sources (river channel and dune sediments) were identified in the field and sub-sampled for particle size distribution and geochemical analysis.

To aid in the identification of lithic sediment sources, the geochemistry of wetland sediment deposits was analyzed for similarity to potential source sediments (fluvial, littoral, dune). Geochemical concentrations were corrected for the influence of grain size, which varies independently of mineral composition, with the latter calculated as the inclusive graphic mean [42] for the elements Al, Ba, Ca, Co, Cr, Cu, Fe, K, Mg, Na, Ni, P, S, Sc, Sr, Ti, V, and Zn. Principal components analysis (PCA) was used to reduce the multi-elemental data matrix to a fewer number of linearly uncorrelated variables through use of a transformation, a method that is commonly used to discriminate between alternative sediment sources [43,44]. For each study site, PCA was conducted on grain-sized normalized elemental concentrations for both processed core and sediment source samples to identify the overall geochemical similarity between core samples and potential sources based on scatterplots of first and second principal component scores. Bartlett’s test of sphericity and the Kaiser–Meyer Olkin (KMO) measure of sampling adequacy were used to identify the appropriateness of the PCA analysis. All sphericity tests were significant; however, for three of the five estuaries, KMO measures were <0.50; in these cases, three elements (with low correlations with other elements) were removed prior to analysis.

#### 2.2.3. Data Analysis

For each lagoon, C storage estimates were made by taking the product of wetland area, soil bulk density, and soil fraction organic C, limited to the first 50-cm of cores only. Values were summed for the five focus sites studied. Estimates of soil organic C storage fluxes were also made by taking the product of wetland area and C storage rate approximates using actual soil organic C density and a minimum accumulation estimate derived from a C storage study conducted in California [45]. To provide insight to factors promoting high soil C, a correlation matrix was used to examine predictors of soil organic C density, including grain size distribution (% clay, % silt, % sand, median size, inclusive graphic mean, inclusive graphic standard deviation) and climatic variables (mean annual temperature and precipitation), and a multiple regression model was fit to predict soil C density as a function of soil texture and climate.

## 3. Results

### 3.1. Analysis of Aerial and High Resolution Satellite Imagery

**Figure 2:**
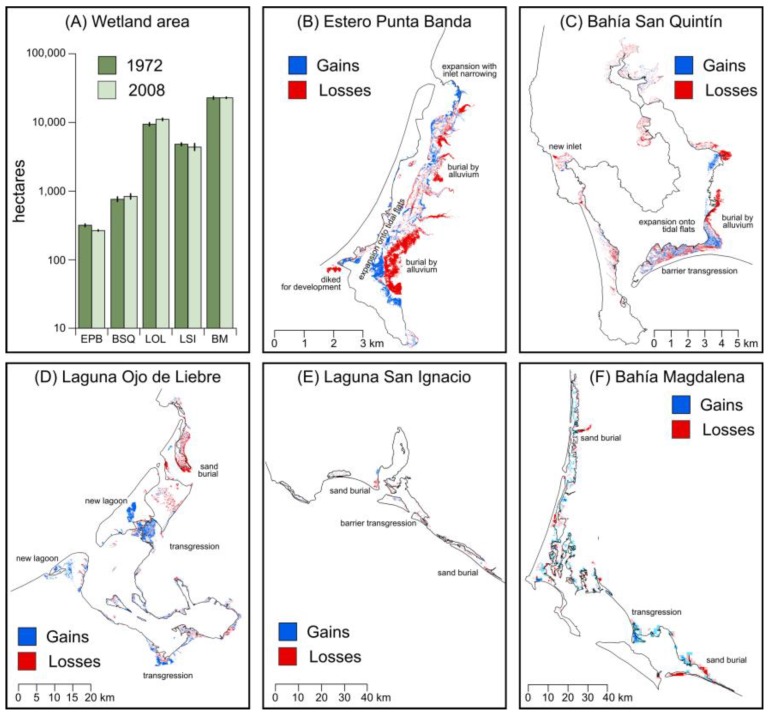
Changes in tidal wetland extent at five Pacific Baja California lagoons: (**A**) an overall gain of 3.9% was estimated, although considerable variability was found from site to site; (**B**) at Estero Punta Banda, wetlands were lost to diking and burial by alluvium; gains occurred as wetlands prograded onto tidal flats; (**C**) at Bahía San Quintín, wetlands were lost to barrier rollover and burial by alluvium; gains occurred as wetlands prograded onto tidal flats; (**D**) at Laguna Ojo de Liebre; (**E**) at Laguna San Ignacio; (**F**) at Bahía Magdalena, dunes buried wetlands, and wetland transgression occurred.

Analysis of aerial photography showed that the extent of tidal wetlands in the five largest estuary/lagoon complexes along the Pacific Coast of Baja California in 2008 approached 40,000 ha, and further suggested that the overall extent of Baja California’s tidal wetlands increased between 1972–2008 (3.9%; Figure 2; Table 1; Supplementary Materials). Net losses were apparent at Estero Punta Banda (−14.5%) and Laguna San Ignacio (−6.6%), while net gains were found at Bahía San Quintín (+10.2%) and Laguna Ojo de Liebre (+16.7%). Little net change was found for Bahía Magdalena (+0.07%).

**Figure 3:**
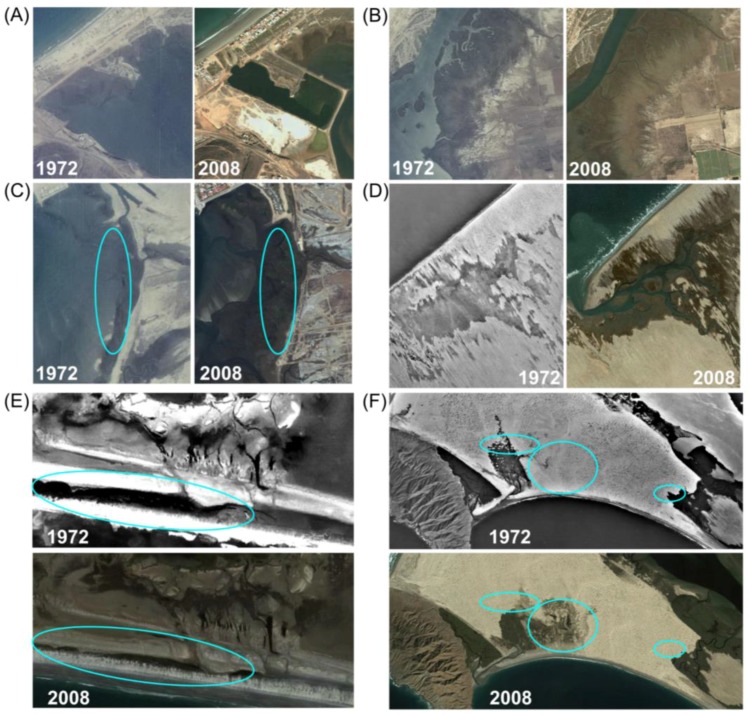
Images of changes in wetland area between 1972–2008: (**A**) at Estero Punta Banda, wetlands were lost to diking; (**B**,**C**) at Estero Punta Banda, wetlands were buried by sediment, and expanded onto former mudflats; (**D**) at Laguna Ojo de Liebre, a new lagoon formed; (**E**) at Laguna San Ignacio, dune rollover buried marsh by sand; (**F**) at Bahía Magdalena, wetland transgression occurred, and wetlands were buried by active dunes. 2008 imagery from Google, Digital Globe.

Examination of aerial imagery revealed the processes responsible for the stability to slight expansion that had been found by image classification. Losses have resulted from coastal development, fluvial sediment burial by prograding river deltas, sand burial by active dunes, barrier transgression, and inlet widening (Figure 3). Gains in wetland areas have occurred along prograding delta fronts, with the formation of a new inlet and lagoon via a barrier breach, and through the transgression of wetland vegetation. Net losses at Estero Punta Banda can primarily be attributed to sediment burial from adjacent creek deltas, and at Laguna San Ignacio to sand burial by dunes. Gains for Bahía San Quintín can primarily be attributed to new wetland formation along the delta front of the Arroyo San Simón. Gains for Laguna Ojo de Liebre can primarily be attributed to the formation of two new back-barrier lagoons.

The comparison of automated to heads-up imagery classification found high levels of agreement between the two methods with respect to areal wetland extent (Table 2). The highest levels of agreement were found for Bahía Magdalena and Estero Punta Banda, the most northerly and southerly lagoons. For nine out of 10 observations, automated classification was found to under-map the extent of mangroves and tidal marshes. Comparing classification uncertainties to changes in wetland extent over time suggests that overall changes of <10% might result from misclassification. However, the substantive changes to mangrove and marsh habitats in Baja California reported by this study can be attributed to dynamic processes of wetland creation and destruction, rather than significant directional gains or losses.

### 3.2. Sedimentary Analysis

**Figure 4:**
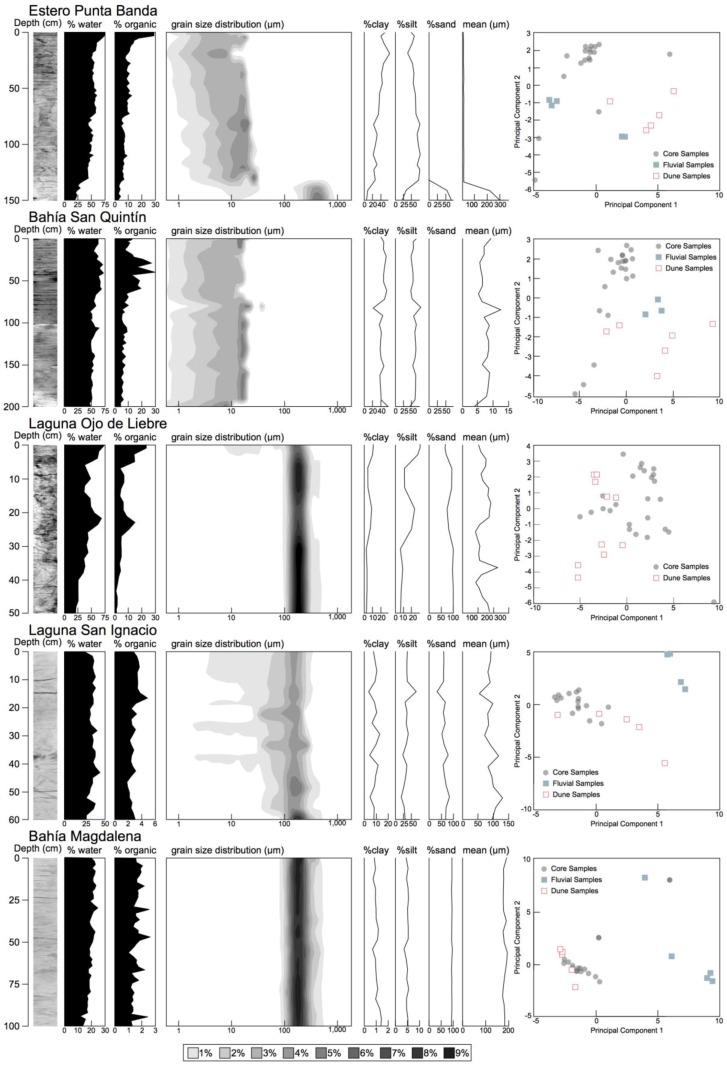
Stratigraphic profiles for cores collected from focus areas. X-radiographs, % water, % organic content, particle size distribution across depths, and results of principal components analysis indicating the geochemical similarity of core sediment samples with potential lithic sediment sources are shown.

The 18 geochemical variables were effectively reduced to two principal components, which explained 61%, 67%, 68%, 69%, and 85% of the variability in the elemental data for lagoons Estero Punta Banda, Bahía San Quintín, Laguna Ojo de Liebre, Laguna San Ignacio, and Bahía Magdalena, respectively. Eigenvalues for the two principal components were 14.0, 14.3, 15.6, 13.8, and 17.0. For the three southerly lagoons, most of the sediment was large-grained (sand sized; >62.5 μm), and the results of the PCA analysis (Figure 4) suggested that the sediments geochemically matched sediment samples that had been collected from active dunes abutting wetlands. For the northerly lagoons, sediment samples were finer grained (lacking sand), and poorly matched both dune and fluvial sediment samples. Clear laminations, or variations in sediment grain size, which are both indicative of episodic sediment deposition events, were not found in x-radiographs or sediment grain size profiles. For Bahía Magdalena, wetland sediments were well sorted, as is typically found for aeolian transported sand; at the four other lagoons, sediments were poorly sorted, as is more typical for fluvially or tidally transported sediment [42]. Analyses show that sediment accumulation for Baja California tidal wetlands occurs primarily as mineral sediment, rather than via peat formation, as values are below the cut-off for organic soils (~20% organic) (Figure 4; Table 3).

A north to south gradient in C density was identified, such that sediments deposited at Bahía Magdalena possessed about one-quarter the C density of the more northerly lagoons (Table 4). Summing the aerial extent of wetlands by wetland C density allows us to estimate C storage for the Baja lagoon wetlands to a depth of 50 cm, at 2.55 Tg (2.55 × 10^12^ g C). Assuming that the Baja California wetlands are accumulating sediment at rates at the low end of those found in other California (United States) wetlands (2.5 mm year^−1^) [45], yearly C sequestered can be estimated at 4.68 × 10^10^ g C year^−1^.

Soil organic C density was found to vary significantly as a function of soil texture and climate (Table 5). Higher soil C density values were found for samples within a higher percentage of clay and silt, and for higher precipitation locales. Lower soil C density values were found for samples with a higher percentage of sand, samples with higher median and mean particle sizes, samples that had been poorly-sorted (high sample standard deviation), and samples at higher temperature locales. The multiple regression model predicted approximately 70% of the variation in soil organic C density (*r*^2^ = 0.698; *p* < 0.001). There was a strong relationship between precipitation and soil texture, such that low precipitation sites had soil with coarser particle sizes.

**Figure 5:**
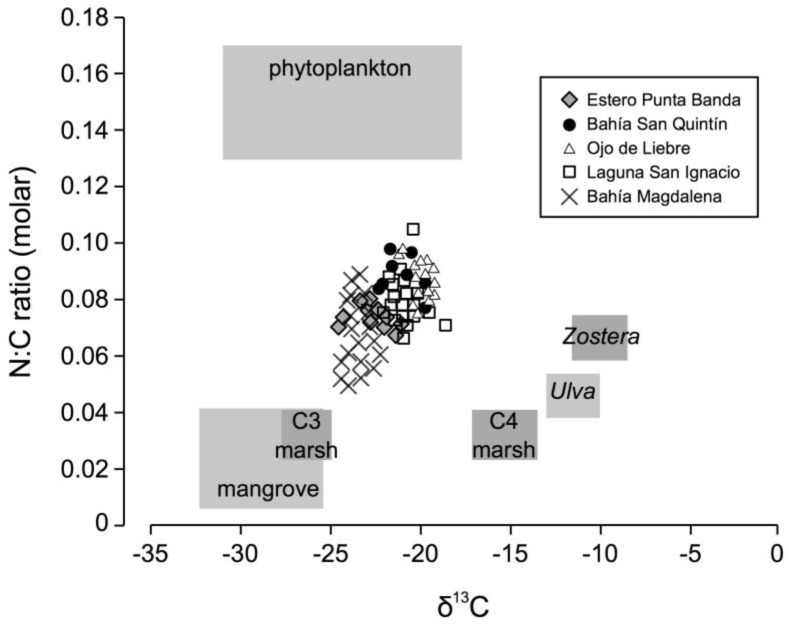
Molar nitrogen:organic C ratios (N:OC) vs. δ^13^C values for wetland sediments from five Pacific coast lagoons. Values of potential end-members are derived from previous studies [3,37,38,39,40,46].

Plotting sediment organic carbon/nitrogen (OC/N) stoichiometric ratios and the stable C isotope ratio (δ^13^C) of the organic material found in sediment cores (Figure 5) shows that C storage in wetlands originates from a variety of sources, and likely includes both phytoplankton and macrophytes. Since potential sources are numerous, a typical or two or three end-member mixing model was not produced, because of the large number of solutions possible for each sample. There was a great deal of overlap in C sources between sites, although sediments from Bahía Magdalena and Estero Punta Banda generally had more of a C3 marsh/mangrove stable isotope signature in comparison with sediments from the other three estuaries.

## 4. Discussion

### 4.1. Carbon Storage

The amount of organic C stored in the top 50-cm of Baja California lagoon wetlands (2.55 Tg) is significant when compared with the yearly estimates of the global coastal marsh C sink (11–87 Tg C year^−1^). Disturbance of these sediments through coastal development, or loss due to erosion linked with sea level rise, could result in the mineralization of buried C equal to 9.36 Tg CO_2_eq, or the yearly CO_2_ emissions of 1.97 × 10^6^ passenger vehicles. Using a conservative accumulation rate of 2.5 mm year^−1^, C storage can then be estimated for these five lagoon complexes at a rate of 12 to 73 g C m^−2^ year^−1^ or 4.68 × 10^10^ g CO_2_eq year^−1^. Given that the areal extent of seagrass found in these lagoons is also significant, and that these seagrass beds likewise capture and sequester significant volumes of C [47], Baja California’s lagoons represent a significant blue C resource, and are especially valuable because its wetlands have been preserved with very little alteration and C mineralization [22]. Recent studies have drawn attention to the valuable ecosystem service provided by the Baja California wetlands in supporting coastal fisheries [48]; our data extends the knowledge of the Baja California coastal ecosystem service provision to C sequestration.

Based on stoichiometry and C stable isotope analysis, this study suggests that the organic C preserved in wetland soils has a combination of sources, which likely include both C3 and C4 marsh plants and phytoplankton, with other sources such as eelgrass and macroalgae also possible. Indeed, during fieldwork, decaying eelgrass mats were commonly observed as wetland wrack deposits. The finding that coastal wetland soils preserve a combination of autochthonous and allochthonous C is in agreement with previous studies conducted using multiple stable isotope and stoichiometric tracers [40,49,50,51]. What is potentially novel about this study is the aridity: watershed particulate C inputs are minimal due to the lack of desert plant cover. It is thus easier to attribute C3 or C4 marsh C sources to wetland macrophytes than in a typical estuary with a forested watershed, which has been a large potentially complicating factor in previous studies [49,51]. In a hydrologically isolated desert wetland, the C source is likely local.

Studies of C sequestration in saline coastal soils report highly variable rates of sequestration globally, spanning two orders of magnitude (ca. 10–1000 g m^−2^ year^−1^) [1]. In addition to sediment deposition rates, which were not addressed by our study, factors relevant for the prediction of C sequestration include mineral surface area, soil organic content, and climate. Studies conducted on organic matter composition and soil texture have determined that larger sediment grains tend to be associated with low soil C density over regions of similar mineralogy [52]. Results presented here include C inventories from coastal wetlands with profoundly different soil qualities that allow us to examine the interactions between sediment grain size and C storage (Table 4 and Table 5). Here, we found a relatively strong relationship between low rates of soil organic C density and coarse sediment texture. Although previous studies have suggested that wetland soils with low organic content tend to have higher bulk densities, evening out the differences between organic and mineral soils, this study instead suggests that given similar accumulation rates, coastal wetlands with organic soils will sequester more C. In addition, these results broadly suggest that climate plays a role in soil C density, with higher temperatures leading to more mineralization, and greater precipitation leading to higher rates of sequestration.

### 4.2. Wetland Stability

This study reports on the soil C density in coastal wetlands that are unusually pristine and have been impacted very little by coastal development and alterations to hydrological processes. We posit that developing a deep understanding of how wetlands are changing over time—in response to coastal development and climate change—is key to determining changes in C sequestration capacity. In addition, tidal wetlands in arid landscapes are not well studied, nor are pristine wetlands. This study thus provides new insights on natural coastal processes that occur in coastal wetlands in arid landscapes, as well as how these pristine wetlands will be affected by sea level rise, therefore identifying potential changes in C storage.

This study reports on wetland change patterns that share both commonalities with and differences from those described for temperate regions of North America. Similar to what has been reported for both the mid-Atlantic and Pacific coast of the United States (U.S.), edge loss has occurred over the past few decades [34,53,54]. Edge erosion is thought to be driven by moderate storms rather than episodic events [55]. Likely, edge erosion is mainly due to natural coastal transgression with sea level, where wetlands along the shore edge are eroded, and new wetlands form along the upland transition zone [25]. However, there is some evidence that wave-driven erosion has increased over time in North America: wetland edges have become smoother over the 20th century [26], which is indicative of greater rates of wave attack [56].

The upslope migration of wetland vegetation due to positive sea level anomalies, which has been noted for Bahía Magdalena by this and a previous study [24], has also been described for the U.S. mid-Atlantic [57], and is predicted to become widespread through this century [58] when not constrained by coastal development [59]. Very gentle wetland–upland slopes are conducive to significant coastal wetland upslope migration, such as found along the Atlantic coastal plain, while steeper topography, such as that found along the Canadian and U.S. Pacific coasts, is much less conducive to marsh transgression with sea level rise. Here, it appears that in Baja California, where coastal wetlands slope gently into strandplains and dunes, there is a great deal of promise for wetland migration and survival with climate change, although there is some concern that ecologically new areas of wetlands do not provide the same level of ecosystem services, including C sequestration, or canopy complexity as wetlands lost along the seaward fringe [24].

Analogous to transgression, this study also identified a significant expansion of wetland areas in central and southern Baja California resulting from the formation of new lagoons (Figure 2). These lagoons appeared to have formed during dune overwash events that resulted in new and stable tidal inlets, leading to the recruitment of wetland vegetation on intertidal islands and along the lagoonal fringe. While new inlets are often cut through barrier islands during storms [60], the formation of new lagoons with coastal transgression is something that has previously only been described for Namibia [61].

Our analyses of stratigraphic profiles also suggest that Baja California Pacific coast tidal wetlands will be relatively resilient to climatic change based on sediment composition and source. Wetlands with mineral soils and little anthropogenic alteration to sediment transport pathways are thought to have low vulnerability to sea level rise. Physical feedbacks promoting sediment accumulation occur when marsh flooding increases, allowing marshes to accumulate at rates similar to that of inundation increases [62,63]. In contrast, where marshes accumulate primarily though peat formation, increased inundation can lead to reduced plant productivity and thus reduced rates of organic matter accumulation [64]. Organic soils were not found in the five lagoons studied, suggesting that in Baja California, peat formation plays a negligible role in marsh accretion with sea level rise. Our examination of sediment grain size and geochemical profiles for several potential sediment sources suggests that the sediment derived from dunes likely plays a large role in sustaining vertical accretion, either through aeolian sediment transport or aeolian sediment re-distributed via tidal sediment fluxes. Further, the mere existence of wetlands among unconsolidated dunes suggests that sediment is abundant.

In summary, this study suggests that the lagoonal tidal wetlands of Baja California appear to have low vulnerability to loss with sea level rise, and as such are an important C storage resource. Wetland sediment accumulation is supported by mineral sediment, and unlike many regions of North America, sediment supply is abundant and not restricted by issues such as upstream dam retention and land-use change [27]. The gentle slopes, relative lack of developed shorelines, and evidence from remote sensing studies all indicate that the environmental context is conducive to upslope migration and transgression. Finally, our analyses documented several instances of wetland habitat expansion linked to the formation of new, hydrologically isolated, back-barrier lagoons through dune breaches, a process that is both relatively novel, and an unconsidered result of sea level rise in arid landscapes. These lines of evidence all suggest that Baja California’s tidal wetlands will be relatively stable in extent with climate change, and will continue sequestering C.

### 4.3. Future Anthropogenic Impacts

Recent publications have highlighted the high rates of C emissions that are associated with accelerated losses of vegetated coastal ecosystems through climate and land-use change [13,65,66]. These ecosystems typically build up over time, storing C in deep organic-rich soil profiles over decades to millennia. When habitats are damaged or destroyed, C locked up in long-term storage is then mineralized to the atmosphere at rapid rates, in pulses that are thought to equal 50 times the annual sequestration rate over a decade [67].

Although this study found little loss of wetlands over the past four decades that could be attributable to anthropogenic impacts, past and future coastal development projects have and will threaten Baja California coastal wetlands and the valuable services they provide, including C sequestration. While the government of México has shown a strong commitment to wetland conservation, applicable protections are not consistently preventing habitat loss [68]. Federal laws and regulations pertaining to biodiversity, wildlife, water resources, and climate change establish guidelines for compatible uses of wetlands and prohibit mangrove destruction [68,69]. México entered into the Ramsar Convention in 1986, and to date has designed 142 sites, the second largest number of any country [70]. In addition, Mexico’s new National Wetlands Policy also explicitly recognized the ability of coastal wetlands to mitigate climate change through C sequestration and their role in reducing emissions from land-use change [71].

However, wetlands remain highly threatened in México despite their protected status. Estimates of potential loss that are based on maps of actual versus potential coastal wetlands have suggested that 62% of Mexican coastal wetlands have been lost due human impacts [19]. In La Paz (Baja California Sur), 23% of coastal wetlands were lost between 1973–1981 to coastal development [48]. Further, despite the presence of legal protections that have prohibited mangrove destruction, mangroves in México are being deforested for coastal development at a rate of 1.1% per annum [72]. Most of the lagoons under study here have been threatened with wetland destruction over past decades; this includes threats from the proposed expansion of commercial salt production to Laguna San Ignacio [73], and the wetland diking that occurred in 1983 for industrial development in Estero Punta Banda [74,75,76]. Baja California’s tidal wetlands have survived in a pristine state because of their isolation, but since the 1970s and the construction of the transpeninsular Highway, they are much more vulnerable to loss from development. Given the high rate of emissions associated with landscape conversion, and the reality that coastal wetland destruction is occurring and will continue in the future, states may consider addressing the inclusion of greenhouse gas mitigation requirements where development impacts coastal wetland habitats.

## 5. Conclusions

Efforts to mitigate greenhouse gas emissions have increasingly focused on preserving and restoring forests and wetlands; as such, these activities provide strong benefits to wildlife and humanity in addition to those of C sequestration. In this study, we summarized the context of C storage functions for the five largest areas of coastal wetlands on the Pacific Coast of Baja California, including land-cover change trajectories and interactions with management and sea level rise. Our remote sensing analysis found a slight increase in wetland extent over past decades (+3.9%), and little evidence for direct anthropogenic disturbance, which forms a strong counterpoint to the history of wetland destruction that has occurred over the past centuries elsewhere in North America. Natural coastal processes unique to arid landscapes drove changes in wetland extent, including the burial of wetlands by active dunes, inlet breaches, and delta front progradation. No evidence for wetland fragmentation and drowning in response to sea level rise was observed, although some upslope migration of mangroves was found, suggesting that inundation increases are occurring. Soil C density was found to vary along the climatic gradient of the Baja California peninsula, with less C dense soils found in southern Baja, in association with warmer and drier conditions, and sandier sediments. We found that disturbances to wetland soils in this region (to a depth of 50 cm) would liberate 2.55 Tg of organic C or 9.36 Tg CO_2_eq. While México has developed strong protections for coastal wetlands, these policies have not been successful at preventing wetland destruction, and Baja California wetlands are very vulnerable to future impacts from coastal development. The continued persistence of these unique landscape features in such an undisturbed state, and the results presented here, argue for the preservation of the current tidal wetland acreage to help mitigate the impacts of greenhouse gas emissions as well as to maintain the other valuable functions these wetlands provide.

## Figures and Tables

**Table 1:** Changes in the areal extent of tidal wetlands between 1972–2008 at five Baja California tidal lagoons. Key drivers of change are listed in order of importance. Factors accounting for losses (−) and gains (+) are described.

	1972 (ha)	2008 (ha)	Change (%)	Key Drivers of Change
Estero Punta Banda	309	264	−14.5	Burial by alluvium (−)
				Recruitment on tidal flats (+)
				Coastal development (−)
				Inlet dimension shifts (+)
Bahía San Quintín	746	822	10.20%	Recruitment on tidal flats (+)
				Burial by alluvium (−)
				Barrier transgression (−)
				New inlet (−)
Laguna Ojo de Liebre	9448	11,026	16.70%	Barrier breach/lagoon formation (+)
				Burial by dunes (−)
				Transgression (+)
Laguna San Ignacio	4690	4379	−6.63%	Burial by dunes (−)
				Barrier transgression (−)
Bahía Magdalena	22,246	22,600	0.69%	Transgression (+)
				Burial by dunes (−)
Total	37,638	39,091	3.86%	

**Table 2:** A comparison of automated and heads-up digitization of mangroves and coastal marsh at five estuaries in Baja California: Estero Punta Banda (EPB), Bahía San Quintín (BSQ), Laguna Ojo de Liebre (LOL), Laguna San Ignacio (LSI), and Bahía Magdalena (BM). Positive values indicate more wetlands were mapped using automated digitization, and negative values indicate more wetlands were mapped using heads-up digitization.

	EPB	BSQ	LOL	LSI	BM
1972	−1.0%	8.3%	−6.2%	−4.5%	−1.4%
2008	−2.9%	−6.7%	−3.4%	−9.9%	−0.8%

**Table 3:** Mean (and standard deviations) of sediment characteristics for focus lagoons.

	Bulk Density g cc^−1^	Clay%	Silt%	Sand%	Graphic Mean (μm)	Graphic SD (Φ)	Organic%	Organic C mg cc^−1^
Estero Punta Banda	0.81	35	64	0	7.5	1.4	8.6	23
	−0.3	−11	−11	0	−2	−0.23	−4.6	−5.3
Bahía San Quintín	0.63	43	57	0	6.3	1.4	9.2	29.3
	−0.13	−7.2	−7.2	0	−1.4	−0.077	−5.4	−2.8
Laguna Ojo de Liebre	0.96	6	16	78	130	2.2	6.4	28
	−0.36	−2.9	−9	−12	−35	−0.6	−5.5	−7.7
Laguna San Ignacio	1.2	8.3	34	58	92	2.6	2.8	4.87
	−0.13	−2.4	−9.5	−9.6	−17	−0.25	−0.63	−0.33
Bahía Magdalena	1.6	1	4.9	94	180	0.76	1.6	6.68
	−0.077	−0.19	−0.51	−0.62	−6.2	−0.035	−0.36	−0.11

**Table 4:** Focus sites, showing locations, tidal wetland extent, mean soil organic carbon (C) density, soil organic C storage (limited to the top 50 cm), and estimated yearly organic C storage in CO_2_ equivalent (accumulation estimated at 2.5 mm year^−1^, a low end estimate based on data from other California lagoons). Two cores were collected per lagoon.

	Location (N/W)	2008 Wetlands (ha)	Soil Organic C mg cc^−1^	Organic C g Lagoon^−1^	Storage g CO_2_eq year^−1^
Estero Punta Banda	31.70°, 116.62°-	264	23	3.04 × 10^10^	7.49 × 10^8^
	31.76°, 116.65°				
Bahía San Quintín	30.35°, 115.92°-	822	29.3	1.20 × 10^11^	2.20 × 10^9^
	30.52°, 116.03°				
Laguna Ojo de Liebre ^1^	27.42°, 113.88°-	11,026	28	1.54 × 10^12^	2.44 × 10^10^
	28.25°, 114.40°				
Laguna San Ignacio ^1^	26.40°, 112.75°-	4378	4.87	1.07 × 10^11^	5.37 × 10^9^
	26.98°, 113.72°				
Bahía Magdalena ^1^	24.50°, 111.85°-	22,600	6.68	7.54 × 10^11^	2.26 × 10^10^
	24.80°, 112.15°				
Total		39,091		2.55 × 10^12^	5.53 × 10^10^

^1^ Analysis of the three southerly lagoons included several wetland complexes located adjacent to—but outside—the hydrologic boundaries of the lagoon.

**Table 5:** Correlation of soil organic C density (mg cc^−1^) with climatic and sediment texture variables (*n* = 75)**.**

Correlation with Organic C Density	*r*	*p*
% clay	0.629	<0.001
% silt	0.374	0.006
% sand	−0.525	<0.001
median grain size (μm)	−0.362	0.007
inclusive graphic mean (φ)	0.557	<0.001
inclusive graphic standard deviation	−0.254	0.046
temperature	−0.493	<0.001
precipitation	0.571	<0.001

## Supplementary Materials

The following are available online at www.mdpi.com/1424-8220/18/1/32/s1, Figure S1: Estero Punta Banda 2008 wetland area, Figure S2: Estero Punta Banda 1972 wetland area, Figure S3: Bahía San Quintín 1972 wetland area, Figure S4: Bahía San Quintín 2008 wetland area, Figure S5: Ojo de Liebre 1972 wetland area; Figure S6: Oje de Liebre 2008 wetland area; Figure S7: Laguna San Ignacio 1972 wetland area; Figure S8: Laguna San Ignacio 2008 wetland area; Figure S9: Bahía Magdalena 1972 wetland area; Figure S10: Bahía Magdalena 2008 wetland area [available as shapefiles].
